# COVID-19 vaccine acceptance and uptake in Indonesia, Nepal, and Vietnam: Key lessons from a qualitative study

**DOI:** 10.1371/journal.pgph.0006381

**Published:** 2026-05-08

**Authors:** Ida Ayu Sutrisni, Vy Pham-Tram, Manish Duwal, Ha Nguyen Thanh, Diana Timoria, Aria Kekalih, Dewi Friska, Ragil Dien, Claus Bogh, Phong Nguyen Thanh, Raph L. Hamers, Abhilasha Karkey, Sonia Lewycka, Mary Chambers, Jennifer Ilo Van Nuil

**Affiliations:** 1 Oxford University Clinical Research Unit Indonesia, Faculty of Medicine Universitas Indonesia, Jakarta, Indonesia; 2 Oxford University Clinical Research Unit, Ho Chi Minh City, Vietnam; 3 Oxford University Clinical Research Unit, Kathmandu, Nepal; 4 Department of Community Medicine, Faculty of Medicine, Universitas Indonesia, Jakarta, Indonesia; 5 Sumba Foundation, Sumba, Indonesia; 6 Hospital for Tropical Diseases, Ho Chi Minh City, Vietnam; 7 Nuffield Department of Medicine, Centre for Tropical Medicine and Global Health, University of Oxford, Oxford, United Kingdom; 8 Oxford University Clinical Research Unit, Hanoi, Vietnam; PLOS: Public Library of Science, UNITED STATES OF AMERICA

## Abstract

Since 2019, the WHO has included vaccine hesitancy among the top ten threats to global health. Additionally, there is global disparity in vaccine availability and access. As part of a mixed methods study that explored COVID-19 vaccine acceptance and access across Indonesia, Nepal and Vietnam from December 2021 to June 2022, we conducted 67 in-depth interviews with purposively selected community members from both urban and rural settings. We used thematic analysis to analyse and interpret the interviews. Dominant aspects that influenced COVID-19 vaccine acceptance in Indonesia, Nepal, and Vietnam included risk perception, trust, logistics and social norms. These aspects were context-specific and fluid as the pandemic course changed over time. Analysis of COVID-19 vaccination rollout in these settings provide prominent lessons reflecting the importance of embracing contextual norms and building and maintaining trust as early as possible for enhanced acceptance of newly introduced vaccines. Understanding and embracing social norms in specific contexts could also support interventions for specific audiences, with more targeted approaches and methods. Continued discussions to delve deeper into contextual social norms and trust is imperative to support acceptance of newly introduced vaccines.

## Introduction

The global response to the COVID-19 pandemic had a large focus on the development and distribution of vaccines, and by December 2020, the first COVID-19 vaccines started to be rolled out on a global scale [1]. In addition to the responses by high-income countries [2], there were also remarkable public health efforts from low- and middle-income countries (LMICs) while often facing healthcare infrastructure challenges and/or resource constraints [3]. As of December 2022, diverse vaccine brands were approved and used across the three countries, 13 in Indonesia, nine in Nepal, and eight in Viet Nam [4].

Despite significant progress, challenges related to vaccine acceptance and hesitancy, as well as access, persisted globally. Vaccine hesitancy, defined as reluctance or refusal to be vaccinated or to have one’s children vaccinated despite the availability of vaccination services [5], has been considered one of the top ten threats to global health since 2019, even before the COVID-19 pandemic [6]. For instance, the current global measles vaccine coverage is declining. Between 2017–2023, coverage for the first dose of measles-containing vaccine declined from 85% to 83%, while the second dose dropped from 74% to 68% [7].

In the context of COVID-19, socio-demographic factors such as gender, age, education, and occupation were shown to influence the acceptance of vaccines [8]. Further, individual and group influences, including trust related to government and authority [9], the perception of one’s own vulnerability, beliefs about vaccine efficacy and safety [8,10], cultural beliefs, and information sources played a significant role in COVID-19 vaccine acceptance [9]. Concerns over the vaccine’s licensing, questions about clinical trials, and development timelines were also highlighted as specific issues [11]. These multidimensional issues, along with interpersonal relationships and institutional credibility, affected trust in the vaccine and impacted COVID-19 vaccination decisions [12,13].

By April 2022, COVID-19 vaccination rates in Vietnam were relatively high (approximately 82.5% of total population aged 12 and up) [14]. However, the country was unable to reach its national target for 100% coverage of COVID-19 booster doses by mid-2022 (38.5% at that time) [11]. The reasons for this gap included the perception of low mortality (from COVID-19) as the pandemic was under control, or scepticism about vaccine efficacy, and finally concerns over the rapid development of the vaccines [11].

In Nepal, a study across urban and rural settings showed that vaccine acceptance was influenced by geopolitical and socio-economic factors [8]. Some individuals stated that they were unvaccinated/partially vaccinated because they were afraid of adverse effects on their future offspring, fertility and/or potential impact on future pregnancies. This stemmed from the exclusion of pregnant women in early clinical trials, persistent dissemination of information/misinformation via social media, perceptions about the vaccine’s country of origin, but also their perceived risk of contracting COVID-19 from the vaccine itself [8]. Despite a low unvaccination rate of healthcare workers (HCWs) (3%), negative perceptions of the vaccines persisted, even among the vaccinated. Similar to Vietnam, despite high initial COVID-19 vaccination rates, the booster coverage in Nepal remained under 15% (as of November 2022). Reported barriers included ineffective advertising, scepticism, inconvenient scheduling, a lack of enthusiastic approach for booster dose campaign and lack of trust [15].

COVID-19 vaccination uptake in Indonesia reached over 86% and 73% for primary doses (dose 1 and dose 2), however booster coverage was much lower, with the uptake remaining under 30% [16]. A cross sectional study conducted in August 2022 showed 93% participants reporting they would accept the next booster doses provided free by the government [17], yet in reality, barriers included concern over its halal content, concerns about side effects and perceived lower risk from the disease meant that booster uptake was low. To understand these dynamics across different settings, we explored the contextual influences that shaped COVID-19 vaccine acceptance and access during a specific pandemic period in Indonesia, Nepal, and Viet Nam.

As part of a mixed methods study, ‘Social Science and Public Engagement Action Research on Covid-19’ (SPEAR) [18], we conducted a qualitative sub-study to explore contextual determinants on COVID-19 vaccine acceptance and access during a specific time period in three LMICs, including Indonesia, Nepal and Viet Nam.

## Materials and methods

### Ethics statement

The study was approved by ethical committees of local host institutes including the National Hospital for Tropical Diseases (Hanoi, Vietnam), the Hospital for Tropical Diseases (Ho Chi Minh City, Vietnam), CDC Daklak (Vietnam), Nepal Health Research Council (Kathmandu, Nepal), Patan Hospital (Lalitpur, Nepal), Faculty of Medicine, University of Indonesia (Jakarta, Indonesia), and the Oxford Tropical Research Ethics Committee (Oxford, UK).

Additional information regarding the ethical, cultural, and scientific considerations specific to inclusivity in global research is included as supporting information (S1 Checklist).

### Study settings

The study teams were located at Oxford University Clinical Research Unit (OUCRU) sites in Indonesia, Nepal and Vietnam. We included a total of 13 study locations across these three countries that were part of the SPEAR study. In Indonesia, sites included the urban settings of Jakarta and Bandung and West Sumba, a rural site. In Nepal, five distinct locations spanning the country’s diverse geographical regions were selected, representing both rural areas (Mustang, Sindhupalchwok districts) which are less densely populated, and urban areas (Bhaktapur, Morang, Patan) with high population density. In Vietnam, about half of the participants were selected from two major metropolis, Hanoi and Ho Chi Minh City as well as rural and mountainous regions, including Nam Dinh and Dak Lak provinces.

In each setting, the nature of the pandemic varied greatly over time, including the case load, the stringency of the public health responses, the nature of media sources, among others. For example, in Jakarta and Bandung, there were higher cases of COVID-19 throughout the pandemic compared with the island of Sumba in Nusa Tenggara Timur [19]. At the start of the pandemic, Vietnam initiated a zero COVID policy and closed borders rapidly, enacting a more stringent response at that time [20–22]. In Nepal, there was an extended nationwide lock down period that was longer than in other settings [23]. Each country also had different dates of vaccine rollout and access to different vaccines at different times, e.g., Indonesia started nation-wide vaccination in mid-January 2021 [24], Nepal started at the end of January 2021, and Vietnam started vaccination in early March of 2021 [11,25].

### Sampling

We used purposive sampling, based on location, gender, profession, vaccine status, education and age to select participants for in-depth interviews. In Indonesia, we also selected participants based on responses from a survey component of SPEAR study. We contacted key informants at study sites prior to data collection to select a list of participants, based on location, gender, profession or work experiences, COVID-19 vaccination status, education and age. We aimed to recruit an estimated sample of 18–24 community participants per country. The researchers in each country compiled lists of potential participants, contacted them via phone, and arranged interviews either online or offline. We sent reminders to the candidates prior to the interview. If the participants did not join the interview, we replaced them with other participants having similar characteristics. For those who did not participate, we did not require them to report reasons for not participating. In parallel with the interview process, we conducted debrief sessions with the research team to discuss highlighted topics from the participants across sites and to refine the interview guide for further topics to explore or when we reached saturation of a topic. We also discussed and refined the sampling frame as needed during these sessions. Recruitment challenges included potential participants not responding to calls, some were no longer willing to participate or they provided invalid phone numbers in the survey. We reminded participants that there were no right and wrong answers during the interview process, interviewers as remained neutral as possible, and valued silent moments to help participants feel comfortable expressing their views. However, if participants directly discussed vaccine misinformation during the interview, we probed on the topic to gather more information and remained as neutral as possible during the interview. At the end of the interview, we addressed misinformation that the interviewee felt comfortable discussing and/or referred participants to discuss with HCWs.

### Data collection

Study investigators developed the semi-structured interview guide, and the study team revised the topics and questions following routine debrief meetings and based on the evolution of the local situation. We explored the following topics: an open narrative of COVID-19 experiences; acceptance and accessibility of COVID-19 vaccines; perceptions, production of vaccines and other related topics (S1 File). The interviews were conducted between December 2021 and June 2022, when the first two doses of COVID-19 vaccine had been introduced across all the sites and the booster doses were underway at some sites. Due to mobility restrictions in these three countries, we conducted the majority of the interviews via phone or audio calls, but we also held face-to-face interviews when feasible.

The researchers provided the participants with details on the study and requested permission to record the interviews. We collected audio-recorded verbal informed consent for those who had a remote interview; those who had in-person interviews were asked to sign a paper consent form. The interviews were audio-recorded, and the interviewers made notes during the interviews.

### Data analysis

The audio-recorded interviews were transcribed in the language spoken and translated into English by qualitative researchers of each country. Handwritten notes were typed and along with other fieldnotes, all files were uploaded into NVivo 12 to support further analysis. The bilingual researchers at each country checked and coded both original transcripts and English translations to ensure consistency.

We had at least one to two coders per country to conduct two cycles of coding on their country’s dataset initially [26]. First, we developed attribute coding (i.e., categorising the transcripts based on demographics data; gender, settings, age, profession, and COVID-19 vaccination status), and provisional coding using open or inductive coding. In the second coding cycle, each analyst compared themes from first cycle to the framework of vaccine hesitancy developed by the World Health Organisation [5]. The framework helped us map potential vaccine hesitancy across the three countries and ensured that we captured the main determinants related to vaccine hesitancy, including contextual influences, individual and group influences, and vaccine specific issues. At the end of this cycle, we had extensive discussions across the sites to organise comparable codes into categories and patterns. We merged datasets from three countries in NVivo 12 and then used a shared Excel sheet and visual thematic mapping to help us understanding the themes and patterns. We continued with more discussions and reflections about the agreements and disagreements across the sites and finally, refined the main themes.

## Results

We interviewed a total of 67 participants, including 39 females and 28 males, from urban (57%) and rural (43%) sites across Indonesia, Nepal and Vietnam. Over 40% of participants were aged 30–39, 31% were aged 20–29 and the rest were over 40 years old. The participants had diverse occupational backgrounds, including professionals, home makers, as well as participants who were unemployed. More than half (55%) were fully vaccinated (2 doses), nearly a third (28%) had received a booster (3rd) shot; 10% were unvaccinated and only 4% of participants were partially vaccinated (1 dose) (Table 1).

**Table 1:** Demographic of participants.

Remarks	Indonesia	Nepal	Vietnam	n = 67
	n = 24	n = 20	n = 23	
**Gender**				
Male	12 (0.5)	10 (0.5)	17 (0.7)	39 (0.6)
Female	12 (0.5)	10 (0.5)	6 (0.3)	28 (0.4)
**Settings**				
Urban	16 (0.7)	12 (0.6)	10 (0.4)	38 (0.6)
Rural	8 (0.3)	8 (0.4)	13 (0.6)	29 (0.4)
**Aged group**				
20-29	7 (0.3)	7 (0.4)	7 (0.3)	21 (0.3)
30-39	9 (0.4)	9 (0.5)	9 (0.4)	27 (0.4)
40-49	3 (0.1)	3 (0.2)	2 (0.1)	8 (0.1)
≥50	5 (0.2)	1 (0.1)	5 (0.2)	11 (0.2)
**Profession**				
Professional, technical	6 (0.3)	7 (0.4)	1 (0.0)	14 (0.2)
Clerical support, service or sales	7 (0.3)	7 (0.4)	8 (0.3)	22 (0.3)
Skilled manual work	2 (0.1)	0 (0.0)	0 (0.0)	2 (0.0)
Unskilled manual work	0 (0.0)	0 (0.0)	4 (0.2)	4 (0.1)
Stay at home/ Housework/Unemployed	6 (0.3)	5 (0.3)	6 (0.3)	17 (0.3)
Retired	2 (0.1)	0 (0.0)	3 (0.1)	5 (0.1)
Student and other	1 (0.0)	1 (0.1)	1 (0.0)	3 (0.0)
**COVID-19 vaccination status**				
Not vaccinated	6 (0.3)	0 (0.0)	1 (0.0)	7 (0.1)
Partially vaccinated (1 dose)	3 (0.1)	0 (0.0)	0 (0.0)	3 (0.0)
Fully vaccinated (2 doses)	11 (0.5)	17 (0.9)	10 (0.4)	38 (0.6)
Fully vaccinated plus booster (3 doses)	4 (0.2)	3 (0.2)	12 (0.5)	19 (0.3)

Based on our analysis, we grouped the findings into four main themes including: risk, trust, logistics, and social norms (Table 2).

**Table 2:** Description of main themes of factors influencing COVID-19 vaccine acceptance in Indonesia, Nepal and Vietnam.

Main themes [5]	Descriptions
Risk	• Fear was the primary driver for accepting COVID-19 vaccines in the early phases of the pandemic. • Perceptions of COVID-19 infection risk varied depending on age, social exposure, certain medical conditions, including comorbidities, pregnancy and breastfeeding.
Trust	• Institution trust was influenced by both current and past experiences with government policies and practices. • Trust in COVID-19 vaccines encompassed concerns about safety, efficacy, vaccine development timelines, and the distinction between immunisation vs vaccination. • Geopolitical trust affected the perceptions of vaccines based on country of manufacture.
Logistics	• Limited vaccine access was more common in rural settings. • Various global rules related to COVID-19 certificates (which vaccines were ‘valid’ to enable entry into a country). • Conflicting times of vaccination schedules. • Complex registration process and requirements (e.g., identity card).
Social Norms	• Fluctuating situations of COVID-19 infection led to reliance on therapeutic and faith-based practices. • Some women still depended on men’s decisions regarding vaccination.

### Risk

Participants spoke about risk when considering whether to vaccinate. On the one hand, respondents spoke about the fear and risk of acquiring COVID-19 infection and the vaccine was viewed as a protection. Many participants who were fully vaccinated, including those who had received a booster shot, often described their motivation for getting vaccinated as predominantly driven by the fear of contracting COVID-19.

“*I was excited and felt happy about the thing [vaccine] that I would get immunity from the vaccine to fight against the scariest disease*.” (P48, 42, male, filmmaker, rural, Nepal)

Similarly, participants with children expressed fear about them being exposed to COVID-19 outside the home, which prompted them to view vaccination as a safeguard for their family members. The fear was fuelled by the media coverage of severe COVID-19 cases, which often led to feelings of worry, motivating them for vaccination.

“*They [parents] still let their children get vaccinated. They were worried because the kids meet a lot of people at school, and they didn’t know what might happen. The kids might only meet their friends at school, but there might be other people around them after school who might be wandering around with COVID. Their parents were worried, so they let them get vaccinated*.” (P35, 27, female, housewife, rural, Vietnam)

On the other hand, the narratives surrounding risk evolved into dialogues about the perceived risk of acquiring infection and, later, the severity of infection. While the first two doses were implemented under the combined policies of travel restrictions and mass vaccination requirements, there was less legislation around the booster doses, and participants’ views about booster doses better illustrated how disease threat and severity were integral to their decision to vaccinate. The perceived sources of risks (or lack of) varied among participants across the sites and were closely related to the country’s situation at the time of data collection.

“*So those who are not forced… who don’t have any job, including me, are not vaccinated until today. My child who does not work in offices is not vaccinated. Except for those who work, it’s hard to be avoided […] because people think the vaccine is not a medication, but it only protects by immunity*” (P3, 70, male, retired, urban, Indonesia)

Another facet of risk was highlighted in participants’ perceptions about the risk of spreading COVID-19 to their family members whom they perceived as vulnerable. For some, the propensity to vaccinate relied on how much risk they would pose to their family members if they became ill. For participants with underlying medical conditions, comorbidities, or pregnant women concerns revolved around severe side effects or long-term health problems from vaccination. In these instances, some decided to postpone their vaccinations, as advised by early vaccination programme instructions, doctors at vaccination centres, or their instincts.

“*I was doubtful at that time because of my own health condition, I had pneumonia. Because I went through that, I was afraid of getting vaccinated*.” (P5, 36, female, non-government organisation, rural, Indonesia)

Additionally, pregnant or breastfeeding women were concerned about the potential consequences for their infants and their health, such as high fever or reduction in their milk supply, which they heard about from others or on social media.

“*At the time of vaccination, I was breastfeeding my son. Initially, we talked about whether I should get vaccinated or not … and deciding it shouldn’t be taken during that time [whilst breast feeding] so I didn’t take the vaccine*.” (P52, 27, female, banker, urban, Nepal)

### Trust

Another critical element that influenced the acceptance of COVID-19 vaccination across the three countries was trust. We found a variety of forms of trust, including trust related to national governments, COVID-19 vaccines, and interpersonal trust [12].

#### Trust in national governments.

One of the factors that influenced participants’ decisions to get vaccinated (or not) was their trust in government policies, as mentioned by a participant from Vietnam.

“ *I felt more secure after that [having vaccine shots]. I mean I completely trust the vaccination policy. When I received the vaccination, I felt much better, […] I don’t know much about the vaccines, but I’ve got to trust the experts and trust the Government. The whole world is doing it, people are getting vaccinated everywhere*.” (P27, 63, male, retired, urban, Vietnam)

As the global response to the pandemic continued, the vaccine mandates persisted, and more vaccine brands gained approval to be used in Indonesia, Nepal, and Vietnam. However, despite these efforts, persistent negative sentiments circulated online, fostering scepticism about the motivations behind COVID-19 vaccination [27,28]. In each country, the government delivery of COVID-19 vaccines was accompanied by state media announcements and extensive public health promotion. The approach to misinformation was decisive in Vietnam, but less so in Nepal and Indonesia where media is not state controlled [29]. Some participants stated that pharmaceutical companies and government authorities benefited from the vaccine rollout. For instance, some participants from Nepal mentioned that vaccines were rushed and commercialized, driven by competition and profits rather than solely for the benefit of public health.

“*This vaccine arrived in a rush with a sense of commercialisation. Some people have also mentioned that it’s a game of money. With many vaccines arriving in the nation, some in my circle have said that it’s become a matter of competition and economics rather than vaccine diplomacy*.” (P54, 34, male, army officer, rural, Nepal)

In addition, the level of public enthusiasm in government programmes was influenced not only by current circumstances but also by participants’ views of service quality at the health centres. Some participants reflected on their past unpleasant experiences at health centres when discussing the COVID-19 vaccination schedule. Participants expressed concerns about long queues, uncertain waiting times and crowded conditions leading to a waning motivation to get vaccinated. Eventually, some participants preferred to prioritise work over vaccine waiting times.

*“People were rarely wanting to get vaccinated, we were queuing, but did not fight over the vaccine. First, because they are lazy to go to the vaccination site. Because they usually see people queuing. Especially in the primary health centres (PHCs). First, they already thought like this “I went to a PHC, I wasted my time leaving my job, and I have to queue but still might not be able to be vaccinated”. It’s not worth it.”* (P24, 34, male, government officer, rural, Indonesia)

#### Geopolitical trust.

In a similar way that participants reported acceptance of vaccination campaigns linked to trust in the governments and institutions rolling them out, we also documented how participants’ perceptions of specific COVID-19 vaccines were also influenced by political affiliations, or trust/mis-trust in the governments of certain countries. For example, the first vaccines available in Nepal were those manufactured in India. Geopolitical tensions between the two nations led to rumours in Nepal about sub-standard vaccines from India, that the vaccines would cause infertility and were being sent to Nepal to supress its population. Participants from all three countries described reluctance or had negative attitudes toward using vaccines manufactured in China because they associated the quality of these vaccines with that of other Chinese products, which they perceived as inexpensive and low quality.

“*Many people say they won’t get Chinese vaccine as it’s not good and instead they will get American or Indian vaccine*” (P57, 57, female, housewife, urban, Nepal)

Once global travel was possible, the origin of the vaccines continued to be an issue for some people. Certain COVID-19 vaccine certificates were not widely recognized abroad. It was mentioned by some participants that the Sinovac vaccine certificate was not accepted by Australia, while the Johnson & Johnson vaccine certificate was not approved by Germany, leading to concern about certain vaccines.

*“Like I said people who got Johnson vaccine can’t travel to Germany and other places and people who got Chinese Verocell can’t go to Australia*”. (P50, 31, male, chef, rural, Nepal)

#### Trust in the COVID-19 vaccines.

For many participants, the issue of trust was towards the vaccines themselves. The concerns tended to relate to mistrust of the expedited vaccine development process, or due to observed or reported side effects. Firstly, the fast-track development of COVID-19 vaccines brought concerns to some participants on its safety and efficacy. Some participants, such as those quoted below, expressed their concerns on the short development time compared to other existing vaccines, which took years to develop and still led to unknown long-term health consequences.

“*Compared to other vaccines which were tested for longer and guaranteed to be safe, like the vaccines that I get for my daughter, which have been in use for years and come with comprehensive statistics on the rates of side effects. COVID-19 vaccines are new, so we can’t tell how good or bad they will prove to be later*.” (P31, 34, female, office worker, urban, Vietnam)

“*I have meet educated people who hesitate to get vaccinated saying ‘this vaccine is developed in less study period and it might have the side effect, instead I will drink milk with turmeric powder.’*” (P48, 42, male, filmmaker, rural, Nepal)

Their personal experiences or stories about others’ experiences ranging from mild to severe side effects or serious incidents, such as mortality after vaccination, prompted doubts about the vaccine’s safety.

“*A person in the neighbouring village was immediately hospitalized because of the vaccine. At that time, they received AstraZeneca, which was said to be very strong. Indeed, in the neighbouring village, someone was admitted to the hospital. Yes. And it also makes the other people afraid to receive the vaccine and don’t want it, they’re afraid, people don’t want that*.” (P20, 24, female, government officer, rural, Indonesia)

However, the perceived risk of side effects had a different impact when the risk from Covid-19 was high, compared to later in the pandemic when infection and mortality rates were dropping. This is illustrated by one Nepali participant as follows:

“*When hundreds of thousands of people started dying of Covid, people in my circle started developing the view that they will take vaccine if it is available to them.” (Interviewer: “Oh, their perception changed?”) “Yes, it changed. Even talking about the booster doses, they are willing to take it as a new variant of Covid is arriving and people still have no permanent solution for it. Now they see it is better to prevent themselves from Covid-19 infection rather than [worry about] the side effects after getting the vaccine*.” (P48, 42, male, filmmaker, rural, Nepal)

Others felt that being vaccinated didn’t necessarily stop reinfection and therefore the booster doses were unnecessary.

“*Though I was vaccinated, I got infected with COVID-19. Now she [participant’s mother-in-law] has the perception that a vaccine doesn’t prevent you from getting an infection so she is unwilling to get the vaccine*.” (P56, 36, female, teacher, urban, Nepal)

In addition, some people perceived that the infections were mild and therefore vaccination is not necessary.

“*I think it was treatment conditions [that matter more in term of tackling this disease]. People are no longer afraid. People where I live get infected, but they still work in the field and weed their field as usual. They still work as usual, instead of lying on a bed*.” (P28, 30, female, office worker, urban, Vietnam)

“*I think it’s [vaccination] not necessary for children. My nieces and nephews and my child, about ten kids altogether, caught it and recovered very quickly. They only had a fever and didn’t need to take fever reducers. Some of them had more severe symptoms and only needed fever reducers, but they still recovered in 3 days. 3 days at most, usually it only took 2 days*.” (P28, 30, female, office worker, urban, Vietnam)

As seen globally, rumours and misinformation about the vaccines were rife in social media and mainstream news and influenced many people’s acceptance of the vaccines, as illustrated in the following quote:

“*Usually people don’t want Sinovac, usually. Yes. ‘I don’t want Sinovac, because Sinovac is bad’, they say. There was news… a student died after being vaccinated. That’s clickbait, it’s just the title. Whereas his death was not due to vaccines. There’s something else. But because people are lazy to read… so when they read the title, they’re already like, wow, after the vaccine, the kid died. If we read the full article, he died because of an accident. It’s viral on social media. So people are scared…I look briefly on social media. I read it right away. We have to read it, read the full article. Sometimes, the parents of students only read the title, they send it to the family group straight away.*” (P4, 27, female, teacher, urban, Indonesia)

As well as misinformation on social media, there were conflicting comments from prominent members of governments or health services which fuelled the public mistrust of the vaccines.

*“Such kind of rumours were there in news. To what extent this news is true or not? But the fact is that it will cause blood clotting in human brain. I have seen such news that Moderna had such side effects. The rumours about the fertility might only be whispers and rumours among the people but I have read about the blood clotting myself in papers... The people and a Prime Minister of that time said that it is better to drink turmeric water*.” (P48, 42, male, filmmaker, rural, Nepal)

Interestingly, in Vietnam, the mainstream media and social media platforms were tightly controlled by the government. Rumours and misinformation was quickly taken down, and the news channels were seen as important sources of accurate information.

Finally, in interviews with participants in Indonesia, we found that the term ‘vaccination’ was less trusted than ‘immunisation’. They distinguished between the two terms, perceiving ‘vaccination’ as referring specifically to COVID-19 vaccines, products manufactured for commercial purposes. In contrast, they saw the term of ‘immunisation’ as a product for health protection, and well known as protection for children.

“*Vaccines are laboratory engineered products that are injected, which have been... according to the information, have been tested and the results are good with a certain percentage. Then it’s injected. They are sold, bought, and put into our bodies. Not through virus attenuation. For immunisation, a weakened virus is injected, mixed with human blood, and injected into the body. Meanwhile, the vaccine is the result of laboratory engineering*.” (P23, 59, male, online driver, urban, Indonesia)

Although participants in Nepal and Vietnam did not explicitly differentiate between the two terms, they distinguished the COVID-19 vaccination from the existing national immunisation programme for children, with concerns about the COVID-19 vaccine safety.

“*Because you have to separate COVID vaccines and the usual vaccines that they are applying for children in mass vaccination schemes, these two are different. The latter has had decades of experience to be manufactured, at the same time they also conducted clinical tests on it, spent enough time, enough factors, enough for them to prove that it was really effective and it was good. Isn’t that right?*” (P45, 63, female, Office worker, urban, Vietnam)

### Logistics

During the COVID-19 vaccine rollout, we identified similar practical issues across the three countries, including inequitable access, issues regarding COVID-19 vaccine certificates, and administrative challenges. Here we present data demonstrating how these issues affected uptake of vaccines.

#### Inequity of access.

Participants, especially from rural areas, expressed a willingness to receive the COVID-19 booster shot but reported challenges in accessing vaccinations. These barriers included a limited supply of vaccines at some health centres. People had to wait and queue for vaccinations, and yet it was not guaranteed that they would receive a vaccination that day. Some community members had to visit health centres multiple times before receiving vaccinations. Participants mentioned that this process conflicted with personal routines such as work commitments. In all the countries, it was particularly hard for low-paid individuals who lost earnings due to missing work.

“*I went to the ‘Puskesmas’ [public health centre], I wasted my time leaving my job, and I had to queue and was not certain to be vaccinated. It’s not worth it. That’s the problem now. People are resentful because when they administer their ID card or family card, they have to queue for hours [..]. After that, they leave, but they still haven’t gotten it [the vaccine].*” (P24, 34, male, government officer, rural, Indonesia)

Others reported that the COVID-19 vaccines appeared to be accessible primarily to individuals with connections to authorities.

“*If people have a ‘network’, they can have easy access to vaccines but for the people with no network, they have to be deprived of it*.” (P55, 24, female, nurse, urban, Nepal)

In Vietnam, in particular, participants also described certain disparities in allocation of COVID-19 vaccination slots in remote areas. For example, clinics in some highland villages had a limited number of vaccination slots available each day due to low supply, which were distinctly prioritised for certain vulnerable groups. Therefore, the vaccines were unavailable for many people who arrived at the clinics without registration or an invitation.

“*Although I wanted to get vaccinated, I could only get that if I was invited because vaccines were scarce at that time… and they arrived only in small batches. Many went to the site but didn’t receive their vaccine, while others had been vaccinated. They feel a bit aggrieved and it made them not want to vaccinate anymore. But after a few days, they still returned to try their luck to get vaccination*.” (P41, 34, female, farmer, rural, Vietnam)

#### COVID-19 vaccination certificate issues.

During the COVID-19 outbreak, across the three countries, vaccination certificates became an essential precondition for various activities, such as traveling, job seeking, supermarket visits and accessing other public facilities. This requirement was reported to shape participants’ propensity to accept COVID-19 vaccines.

“*I’ve been vaccinated, so I’m safe, I don’t have any problems when I want to go with my vehicle, take public transportation, take a train, or take a plane from Surabaya. There are no problems because I already have a vaccine certificate in the “Peduli Lindungi” [the national COVID-19 mobile application].”* (P13, 53, female, office worker, urban, Indonesia)

“*I had a job before COVID, I used to work as an accountant but I lost my job due to COVID. When I applied for my next job after a year, at that time I had not got the vaccine and they asked whether or not I was vaccinated. Priority was given to those vaccinated.”* (P53, 26, male, unemployed, urban, Nepal)

As discussed above, in addition to national restrictions, the origin of the vaccine meant that some vaccine certificates were not globally accepted, leading to logistical challenges for participants with those certificates.

#### Administrative challenges.

To facilitate the implementation of COVID-19 vaccination in local areas, several vaccination centres distributed online registration links for COVID-19 vaccination through social and communication media platforms. Despite these efforts, some participants encountered registration challenges, particularly with people and groups that had lower access to technology such as the elderly and some rural communities. Participants from Nepal, for instance, reported missing the registration via the online system.

“*At the vaccination centre, they told people to visit there only after completing the online registration. Lots of people missed getting vaccinated as they are not capable of filling out the online form*.” (P56, 36, female, teacher, urban, Nepal)

In addition, participants also reported that community members who were not registered in their local municipality health system were unable to access the vaccine. In Vietnam, economic migrants struggled to get vaccinations if they were living outside of their home province where they are registered in the health system. Due to strict travel restrictions they were unable to return home and were unable to access vaccines in their temporary location.

In rural areas in Indonesia, access issues affected individuals who did not have an identity card, which was a prerequisite to get the COVID-19 vaccines.

“*Some residents don’t have an ID number, that’s why they’re not included, they’re not entered in the system. That’s the problem now… because people do not have an ID number*.” (P24, 34, male, government officer, rural, Indonesia)

### Social norms

Lastly, social norms and practices influenced COVID-19 vaccine uptake and/or hesitancy, including traditions to rely on alternative remedies when dealing with common infections and gender influences in decision-making. Some of the social norms that we identified were country-specific. For some participants, their religion influenced their social norms, such as those from Indonesia who referred to their religious faith as an alternative approach to their healthcare practice.

“*Well, my wife was strong. We slept together. I don’t know what power she has, she didn’t get infected. Yes. But she is diligent in reciting the Quran. That’s probably the religious approach. Well, that’s my wife. My grandson also slept with the one with COVID, he was okay as well. Yes, only 1.5 years old, he was okay. If it’s time to die, then let it be*.” (P23, 59, male, taxi driver, urban, Indonesia)

Additionally, it was important for vaccines to have halal status for some Indonesian participants, particularly when people were making decisions whether to have COVID-19 vaccine booster shots or not. The halal issues were predominant when more vaccines were available to the public rather than in the early pandemic stages since a fatwa of the Indonesian Ulema Council or in Indonesian called Majelis Ulema Indonesia (MUI – Indonesia’s highest religious body) had stated: “In an emergency, it is permitted to do what is normally prohibited” [30], thereby allowing the Muslim population to accept non-halal vaccines, such as the Sinovac vaccine which was the first vaccine available in the country. In the later stages of the pandemic, it was perceived as less of an emergency situation. The halal concern was a dominant driver identified in the urban study sites of Jakarta and Bandung where the population is predominantly Muslim, but not so in the rural Indonesian study site of Sumba, where the population is predominantly Christian.

*“I’m currently considering whether to take it [the booster dose] immediately or not, I’m sorry… related to the halal status. That’s one of the things I consider, apart from the types. Even though to my knowledge, there are only a few vaccines that have been certified as halal. Even though it is currently being promoted, what I know is also for boosters, Pfizer, and AstraZeneca, to my knowledge it’s not halal yet.”* (P11, 31, male, teacher, urban, Indonesia)

Similar religious concerns were not mentioned by participants in Nepal and Vietnam.

For some participants, their approach to COVID-19 was influenced by their usual healthcare practises, particularly if their experience of COVID-19 infection was mild. They likened it to common seasonal infections for which they normally self-treat, and therefore they did not seek vaccination for COVID-19. In addition, after the peak of the pandemic and with fewer severe cases, people returned to their social norms for treatment of illnesses.

“*Two aunties have not got it [vaccines]. (I: Yes) Saying this is just a common cold so they don’t wear masks. There are people like this here who are not getting vaccinated. Well, a lot of (diseases) came and go. They have not got vaccinated saying they are healthy, they won’t wear mask and won’t take medicine for the simple common cold.”* (P48, 42, male, film maker, rural, Nepal)

*“She [participant’s wife] followed me. When she wants to go to the doctor [to get the COVID-19 vaccine], I said why go to the doctor, there is herbal medicine [laughs]*.” (P3, 70, male, retired, urban, Indonesia)

## Discussion

Generally, our findings on COVID-19 vaccine acceptance align with current literature [5,12,31–33], however, specific factors were profoundly pointed out by the sociocultural and COVID-19-specific contexts of the three study countries during the data collection period. It is important to note that these findings are based on particular moments in a pandemic trajectory that, as described above, was very different across the study countries and even between individual study sites in one country. The main factors include social norms, trust, risk perceptions and logistics. Our analysis identifies considerations for improving public health, community engagement or other vaccine-related interventions including the need to understand and work within social norms, and the importance of fostering public trust in vaccination programmes. We suggest that trust is strengthened through improving delivery of care (logistics), and clear communications of risk.

### Embracing social norms

The communities in our study functioned within the existing social norms to respond to the outbreak, which influenced their motivations and decisions about COVID-19 vaccines. For example, some participants opposed the COVID-19 vaccines, yet at the same time, expressed a positive attitude towards immunisation programmes for children. This might be explained by the idea that positive attitudes toward vaccines do not always translate into practice [34]. However, adult immunisations are generally not included in national programmes, except for tetanus immunisation for pregnant women. This could contribute to the common paradigm that immunisation is given during childhood.

Another norm was that people were unlikely to accept something “new”. This highlights that vaccine mandates, either implicit or explicit, need to be carefully considered in vaccination policy. Although vaccination uptake might increase, it might also create scepticism [35]. Given that vaccination is also dynamic [36], strengthening the visibility of pro-vaccine actors beyond the government [34] may support collective action and build more proactive stories while allowing individuals time to process and accept vaccination. This is particularly relevant in both outbreak and non-outbreak settings, where the protective value of herd immunity may be perceived as an abstract concept or an individualised benefit, for instance, in the decline of booster uptake following the decrease in COVID-19 cases. In addition, some viewed immunisation and vaccination as distinct concepts, with vaccination perceived as a ‘new’ development emerging during the COVID-19 pandemic. We argue that improving communication and awareness about vaccines [32,33,37], and immunisations is essential to improve vaccine acceptability and uptake in the local context [38].

In Indonesia, religion is an important social norm, and religious considerations can have a prominent influence on vaccination programmes, such as the refusal of the measles rubella vaccine campaign in 2018 [39]. It was concluded that the Sinovac type was halal [40], while the AstraZeneca vaccine used trypsin derived from porcine in the manufacturing process, but it was still allowed to be used in the context of an emergency situation [40,41]. However, even within Indonesia, this norm is context-specific and it was mainly discussed by participants in urban areas with a Muslim majority and less mentioned in the rural site, Sumba, which has a Christian majority. Therefore, in Indonesia, religious leaders are an important group to be engaged with when introducing new vaccines.

Although we did not approach the topic through a gender lens, as it was beyond the scope of the initial study aims, the influence of gender on vaccine acceptability was a norm in certain settings— some women’s decisions depended on the approval of men (husbands) for taking COVID-19 vaccines. Gender can also influence other health issues in these countries—for instance, in reports of leprosy cases in Nepal and Indonesia cultural traditions often require women to depend on their husbands or in-laws for decisions [42]. Similarly, some women need their husband’s permission to use contraception, demonstrating a lack of freedom for women on health choices [43].

In the third phase of the pandemic, there was a decline in severe cases of COVID-19 across the three countries. Although the respective governments continued their vaccination programmes, there was a waning in public motivation to accept booster doses. Sustained engagement and understanding of the social norms in each context is essential to increase motivation towards vaccines and foster trust [38].

### Fostering trust

There is substantial recent research examining how trust influences acceptance of public health control measures [44,45]. The literature on COVID-19 vaccine acceptance particularly identified distinct types of trust: trust in institutions, including governments and healthcare providers and also trust in the COVID-19 vaccines [12]. Understanding different drivers of trust are essential to design effective approaches to increase and maintain vaccine acceptance.

In terms of institutional trust, a global study reported that citizens in Vietnam have the highest levels of confidence in national health services [46], leading to strong compliance with the government policies, resulting in high rates of acceptance of the COVID-19 vaccine. This aligns strongly with our data. We conclude that trust is built by the public’s experience of a competent health service, as seen in the rapid response to the first COVID-19 cases in Vietnam, but it is also likely that the public remembers the Vietnamese government’s effective control of the Severe Acute Respiratory Syndrome (SARS) epidemic in 2003 [47,48]. Similarly, a study from Nepal found a significant association between perceptions of health system performance and local-level trust in Nepal, demonstrating that service delivery and performance are direct drivers of political trust [45]. In contrast, the eroding of trust in institutes and state services is compounded by low satisfaction in the health services, such as long waiting times, [49] uncertain schedules and unequal access [50]. We conclude that addressing logistical and administrative challenges would have improved uptake not only to the COVID-19 vaccines, but also to other life-course vaccinations provided for the communities. The second focus of trust was on the COVID-19 vaccines themselves. Although not all views from the participants were negative, it is important to note that geopolitical prejudices could hinder acceptance of certain vaccines.

Finally, trust in states and institutions can also be eroded when people are exposed to inconsistent information in the media [51]. In the same way, lack of transparency about vaccine development and research drove mistrust [17,52]. Mainstream media in Vietnam is state-controlled, and was seen as a reliable source of information during the pandemic [47]. On the other hand, analysis of media in Indonesia during this time revealed a lack of transparency in the dissemination of COVID-19 information and data in Indonesia driving mistrust in the government’s handling of the pandemic [53]. Although the abundance of online media enables the rapid circulation of misinformation and rumours, there is also a correlation with digital literacy and acceptance of disease control measures. A study on media literacy in Vietnam showed a positive correlation between young people’s media literacy and their trust in government, compliance with public health regulations, and vaccine acceptance [54]. A study in Indonesia reported that hesitancy towards booster doses in Indonesia was linked to lack of knowledge, including about side effects, halal status and benefits [17]. Our data similarly showed that lack of information and knowledge about the vaccines and the government control measures led to mistrust in the government and the vaccines, and hesitancy to accept vaccination.

These findings highlight the need for transparency from governments and health providers in times of public health emergencies, with more focused campaigns and communication that build the public’s knowledge about research and vaccines and sustain trust in health authorities and hinder spread of misinformation.

## Limitations

This study was limited by the social mobility restrictions in place during the data collection phase. Hence, most the interviews were conducted online leading, in some instances, to technical problems due to poor connections and background noise. These interviews were likely shorter because the interviewers did not have sufficient time and opportunity to build rapport, as they would during in-person interviews. There were also challenges during the participant recruitment process, which took place mostly online and through our established research networks. One important recruitment challenge was the difficulty in recruiting community members with extreme attitudes of vaccine hesitancy; therefore, data from these community members is missing from our analysis. Another important limitation is that the themes related to gender norms and decision-making were developed during the analysis process and therefore we were not able to probe more on these topics with additional data collection; this may have affected the richness of the data. Finally, due to the contextual specifics of each country and the nature of the pandemic at the time of data collection in each site, there may be some limits to the cross-country comparison and its use in future pandemics.

## Conclusions

In summary, the dominant aspects that influenced COVID-19 vaccine acceptance in Indonesia, Nepal, and Vietnam were social norms and trust. We reflect on the importance of understanding the social norms to develop interventions that meet the needs of specific populations and communities. Trust was found to be driven by perceptions of the government’s handling of the control responses (logistics). Perceptions of risk were influenced by the availability of accurate, timely and transparent communications about the vaccines and the public health measures. These factors were all context and temporally specific and could change based on the context or during the course of a pandemic. We also conclude that vaccine acceptance relies on trust, which should be built and maintained prior to a public health emergency. This trust depends on the publics’ experience of reliable and responsive health services, and transparent and accessible communication about research, vaccines and public health measures. Communication should be tailored for local contexts and social norms.

## Supporting information

S1 File Interview guide in English. (PDF)

S1 Checklist Questionnaire on inclusivity. (DOCX)
