# Supplementary material for: COVID-19 vaccine acceptance and uptake in Indonesia, Nepal, and Vietnam: Key lessons from a qualitative study
Source: PLOS Glob Public Health. 2026 May 8;6(5):e0006381. doi: 10.1371/journal.pgph.0006381 (PMC13155667; doi:10.1371/journal.pgph.0006381)
Supplement: S1 File — (PDF) [file pgph.0006381.s001.pdf]

Semi-structured interview – Vaccines related component (community)

In-person interviews will last between 1-2 hours; online interviews may be conducted over a series of shorter online interactions with the same participant. Questions might be re-phrased, when necessary, and additional topics and probes will be included, based on the responses of the interviewees, as well as information gathered during other data collection.

In general, all questions in this guide should be asked, however, the order can be edited to make the interview more conversational.

Ice breaker
Informed consent<br>If first interview, conduct full consent process; if subsequent interview, remind participant about nature of study and consent process. In all cases, ask participant if they have any questions about the study or their participation in this study.
Participants' information
Participant ID<br>Age<br>Occupation<br>Gender<br>Have you been vaccinated? Yes – No<br>If yes, are you fully vaccinated or one dose?<br>Which type of vaccine?<br>When did you receive your most recent vaccine dose?
Interview part 1: Open ended narrative of COVID-19 experiences
[*Goal: to gain a narrative of experiences from the participant's point of view prior to any focused questions to gain a picture of what their experiences include.]*<br><br>1. ***[if vaccinated/if not yet vaccinated*]** To start, we would like to know more about your experiences with getting your COVID-19 vaccine/related to COVID-19 vaccines. After, we will ask more detailed questions but to begin, please tell us about your experiences with COVID-19 vaccines. Please start the story where you like and take as much time as you need.<br><br>2. [*when they are finished*] Thank you for sharing your story. I would like to ask you more about [*insert 1-2 questions that you would like to probe on from their story, if anything in their narrative is related to vaccination, probe here*]. Probe: Are there any obstacles or challenges getting the COVID vaccine? Are there any conveniences that are felt because you have been vaccinated?<br><br>[*probe: during the narrative, try not to interrupt for details – note any questions you would like to probe on, use acknowledgement probes, e.g. ok, yes, mhhh and gentle probes, e.g. "is there anything else you would like to add" or "what else happened?" and then ask follow-up questions.]*
Interview part 2: Perceptions of vaccines
We would now like to ask some questions regarding COVID-19 vaccines.

3. Could you tell me more about what you know about COVID-19 vaccines?
    a. [*potential probes: what is vaccination? How does vaccination work? How many covid-19 vaccines do you know about? Add additional probes from survey results/key informant discussion]*
4. What have you heard from your family, friends, and neighbors about COVID-19 vaccines?
    a. What are the general perceptions of these people toward vaccination?
    b. Are people talking about vaccines a lot? If yes, what are they saying?

5. What do you think about COVID-19 vaccination?
    a. Have your (or other people's) perceptions about COVID-19 vaccination changed over the past year? If yes, please explain.
    b. [*if not discussed*] Do you think vaccines are effective to slow the pandemic?
6. What do you think about vaccinating children with COVID-19 vaccinations?
    a. Probe by type of vaccine, age groups (6+, 12+), number of doses, etc.
    b. What do people in your community think about vaccinating children?

7. Have you heard about COVID-19 booster doses? If yes, what do you think about it? If no, [briefly explain and ask what they think].

8. Is there anyone you know or have heard about who has expressed hesitancy regarding COVID-19 vaccines? Why? [probe for details – probe for hesitancy about childhood vaccination and/or booster vaccines/Religious concerns/pregnant women or women who want to get pregnant concerns re covid vaccine/COVID vaccine types]

9. Have you heard any rumors about COVID-19 vaccines?
    a. [*probe for details on specific situations, for each rumor]* Where did you hear of it? How much do you believe this? From where do you think this rumor started? Do you think these rumors affect people's decision to get vaccinated? Why or why not?

10. What do you think you should you do to protect yourself and your family during this outbreak or future outbreaks? [*probe: for each method, ask their perception of the effectiveness and how easy or difficult it is for them and their family to do it and why*]
    a. [*adjust based on context*] How will this change after vaccines are widely used in your context?

Interview part 4: Vaccine production and access

11. Do you think vaccines coming from different countries are different? Why or why not? If yes, in what ways are they different?
    a. [*if multiple vaccines available or perceptions about certain vaccines are negative*] Do you think people should be free to choose which type of vaccine they receive?

12. [*VN specific question*] Have you heard about the vaccines being developed in Vietnam? If yes, what have you heard about it?

13. What have you heard about the vaccine distribution plan of the government? What do you think about it? Any ideas on how there could be more effective distribution and/or access?

14. What do you think about requiring COVID-19 vaccination for travel or for entering shopping centres?

15. Are vaccines available to you and your family now?
    a. [*if not vaccinated or someone in their family*] Do you plan to be vaccinated? Why or why not?

16. [*add country-specific questions, as appropriate*]
    a. [*Indonesia*] Could you tell us lessons learned (e.g. success stories) of covid vaccine acceptance in Indonesia?[Probe: Motivating and hindering factors]
    b. [*Indonesia – if not covered in subsequent questions*] How easy do you get COVID vaccine? Preference type of COVID vaccine: Sinovac is higher than moderna/Pfizer alike. ---→ was that true? Change genetic code reason? or infertility?

Interview part 5: Wrap up

17. Is there anything else you would like to add to our discussion related to your experiences of COVID-19 and/or COVID-19 vaccination?
